# RANK Expression and Osteoclastogenesis in Human Monocytes in Peripheral Blood from Rheumatoid Arthritis Patients

**DOI:** 10.1155/2016/4874195

**Published:** 2016-10-16

**Authors:** Yuki Nanke, Tsuyoshi Kobashigawa, Toru Yago, Manabu Kawamoto, Hisashi Yamanaka, Shigeru Kotake

**Affiliations:** Institute of Rheumatology, Tokyo Women's Medical University, 10-22 Kawada-cho, Shinjuku-ku, Tokyo 162-0054, Japan

## Abstract

Rheumatoid arthritis (RA) appears as inflammation of synovial tissue and joint destruction. Receptor activator of NF-*κ*B (RANK) is a member of the TNF receptor superfamily and a receptor for the RANK ligand (RANKL). In this study, we examined the expression of RANK^high^ and CCR6 on CD14^+^ monocytes from patients with RA and healthy volunteers. Peripheral blood samples were obtained from both the RA patients and the healthy volunteers. Osteoclastogenesis from monocytes was induced by RANKL and M-CSF* in vitro*. To study the expression of RANK^high^ and CCR6 on CD14^+^ monocytes, two-color flow cytometry was performed. Levels of expression of RANK on monocytes were significantly correlated with the level of osteoclastogenesis in the healthy volunteers. The expression of RANK^high^ on CD14^+^ monocyte in RA patients without treatment was elevated and that in those receiving treatment was decreased. In addition, the high-level expression of RANK on CD14^+^ monocytes was correlated with the high-level expression of CCR6 in healthy volunteers. Monocytes expressing both RANK and CCR6 differentiate into osteoclasts. The expression of CD14^+^RANK^high^ in untreated RA patients was elevated. RANK and CCR6 expressed on monocytes may be novel targets for the regulation of bone resorption in RA and osteoporosis.

## 1. Introduction

We previously reported that the expression of receptor activator of NF-*κ*B ligand (RANKL) on activated T cells from rheumatoid arthritis (RA) patients is elevated [1]. Atkins et al. [2] reported that CD14^+^ receptor activator of NF-*κ*B (RANK)^high^ cells constitutes a circulating preosteoclast pool. The detection of committed pre-OC in peripheral blood is important in the management of disease characterized by abnormal osteoclastic activity. Kochi et al. [3] reported that CCR6, a surface marker of Th17 cells at 6q27, is critically involved in IL-17A-driven autoimmunity in human diseases and associated with RA. Th17 cells express both CCR6 and CCL20. CCL20 is also expressed in fibroblast-like synoviocytes (FLS) from RA patients [4]. In this study, we hypothesized that the expression of CD14^+^RANK^high^ in nontreatment RA is elevated and that CCR6^+^RANK^+^ monocytes migrate into the joints where CCL20 production is elevated. These monocytes differentiate into osteoclasts due to the presence of RANKL expressed on synoviocytes. Thus, monocytes expressing both RANK and CCR6 play a pivotal role in the joint destruction of RA.

Our findings suggest that monocytes expressing both RANK and CCR6 play a pivotal role in the joint destruction of RA and osteoporosis.

## 2. Methods

### 2.1. Study Population

Four untreated and seven treated RA patients (age: 40–59), 6 healthy volunteers (HV) (age: 26–50), and 10 osteoarthritis (OA) patients (age: 57–70) were enrolled. None of them had been taking corticosteroids. All RA patients met the American College of Rheumatology criteria for RA [5]. RA patients were treated with disease modifying antirheumatic drugs, that is, methotrexate.

### 2.2. The Expression of CD14^+^RANK^high^ and CCR6 on Monocytes

Antibodies were purchased from companies as follows; FITC conjugated anti-CD14 (clone M5E2, BD Biosciences, San Jose, CA, USA), PE-conjugated anti-human RANK (clone 9A725, IMGENEX, Littleton, CO), and Alexa Fluor^©^ 647 anti-CCR6 (clone 11A9, BD Biosciences). Peripheral blood samples were obtained from RA patients and healthy volunteers.

To study the expression of RANK and CCR6 on monocytes, three-color flow cytometry was performed. 100 *μ*L aliquots of blood samples were stained with fluorescence conjugated antibodies or isotype-matched mouse IgG controls. After RBC lysis, cells were analyzed using the FACS Calibur flow cytometer (BD Biosciences) and FlowJo software (Treestar Inc., Ashland, OR, USA).

### 2.3. The Mean Fluorescence Intensities (MFI)

MFI was calculated using the intensity of fluorescence of RANK and the number of CD14^+^RANK^high^ monocytes. Thus, MFI represents the mean level of RANK fluorescent intensity of monocytes.

### 2.4. Cells and Cell Culture for Osteoclastogenesis

Peripheral blood samples were collected from the patients with RA and HV. Peripheral bold mononuclear cells (PBMCs) were isolated by centrifugation over Histopaque 1077 (Sigma, St. Louis, MO, USA) density gradients and washed and then resuspended at 1.3 × 10^6^ cells/mL in alpha-minimal essential medium (GIBCO BRL, Gaithersburg, MD, USA) supplemented with 10% fetal bovine serum (JRH Biosciences, Lenexa, KS, USA). Then, PBMCs were cultured for 3 days in 48-well plates in the presence of M-CSF (100 ng/mL). Formation of osteoclasts was evaluated by immunohistological staining for vitronectin receptors after culture in the presence of sRANKL and M-CSF for 7 days. Then, areas of osteoclasts in all wells were evaluated.

## 3. Results

### 3.1. The Expression of CD14^+^RANK^high^

**Figure 1:**
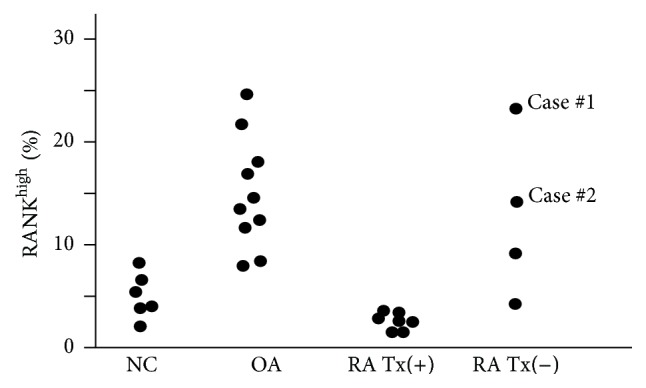
The rates of RANK^high^ in PB. *y*-axis shows % of RANK^high^. Each black circle represents the levels of each of the patients or normal volunteers. RA patients were treated with disease modifying antirheumatic drugs, that is, methotrexate.

The rate of the expression of CD14^+^RANK^high^ on monocytes in untreated RA patients was elevated compared with those receiving treatment. The rate of CD14^+^RANK^high^ expression on monocytes in untreated RA was 4.5 to 24%; in treated patients was less than 4.5%; and in HV was 3 to 10% (Figure 1). Using Spearman's test, we studied the relation between age and RANK; age and RANK were significantly correlated (*p* = 0.0027) (data not shown). OA patients are older; thus, the levels of RANK are high in OA patients.

**Figure 2:**
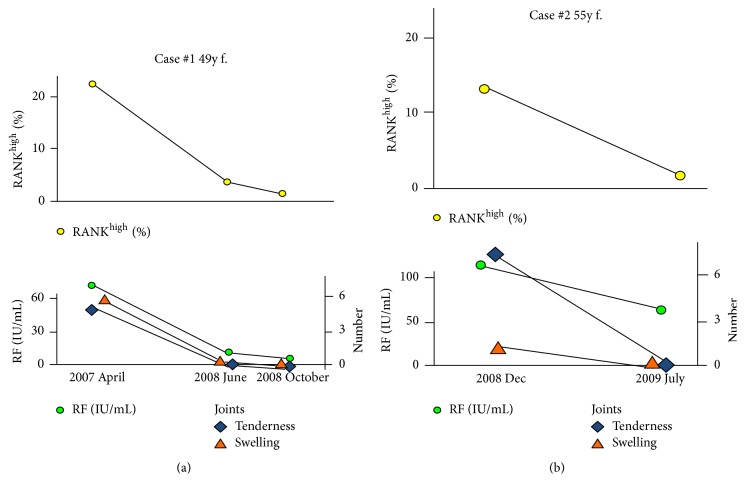
(a) RANK^high^ and RF in case 1. Upper graph shows the decrease of the ratio of RANK^high^ on monocyte during the clinical course. Yellow circle represents RANK^high^. Lower graph shows the decrease of RF level, joint tenderness, and joint swelling during the clinical course. Green circle, orange triangle, and blue diamond represent RF, joint swelling, and joint tenderness, respectively. (b) RANK^high^ and RF in case 2. Upper graph shows the decrease of the ratio of RANK^high^ on monocyte during the clinical course. Yellow circle represents RANK^high^. Lower graph shows the decrease of RF level, joint tenderness, and joint swelling during the clinical course. Green circle, orange triangle, and blue diamond represent RF, joint swelling, and joint tenderness, respectively.

The rate of the expression of CD14^+^RANK^high^ on monocytes was decreased with disease improvement after treatment with DMARDs in RA patients. Figures 2(a) and 2(b) show rheumatoid factor (RF) and CD14^+^RANK^high^ on monocytes of case #1 and case #2 during the clinical course, respectively. Both RF and CD14^+^RANK^high^ on monocytes decreased in parallel with the disease activity of RA in these patients treated with disease modifying antirheumatic drugs (DMARDs).

### 3.2. The Correlation between Osteoclastogenesis from PBMCs and MFI of RANK Expressed on Monocytes from HV

**Figure 3:**
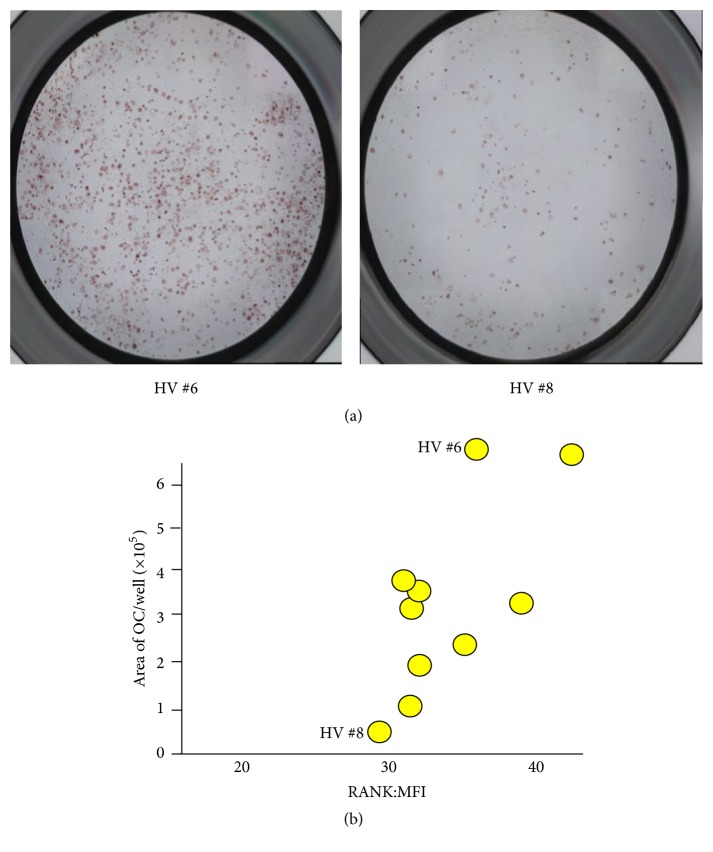
(a) Representative osteoclastogenesis in 2 HV. The left figure shows osteoclastogenesis of  HV #6. The right figure shows osteoclastogenesis of  HV #8. (b) Osteoclastogenesis and RANK. The total area of OC from PBMC was significantly correlated with MFI of RANK. *p* = 0.011. *x*-axis shows the levels of RANK MFI of monocytes. *y*-axis shows the area of osteoclast per well (×10^5^). Each yellow circle represents each HV.

The level of osteoclastogenesis largely varied among HV. Total area of OC from PBMC of HV was correlated with MFI of RANK.  Figure 3(a) shows the representative osteoclastogenesis of 2 HV, a strongly induced one (HV #6) and a weakly induced one (HV #8).  Figure 3(a) is one well of a 24-well plate. We made one-well picture using BZ 9000 one-box microscope of KEYENCE Co. Therefore, osteoclasts are mature although they looked like small immature osteoclasts. We wanted to know the number of osteoclasts, so we stopped the culture before osteoclasts fused into larger size to count the number of osteoclasts.

Figure 3(b) shows the correlation of osteoclastogenesis and RANK. As shown in Figure 3, the total area of OC from PBMC was significantly correlated with MFI of RANK (*p* = 0.011).

### 3.3. The Expression of RANK and CCR6 on Monocytes

**Figure 4:**
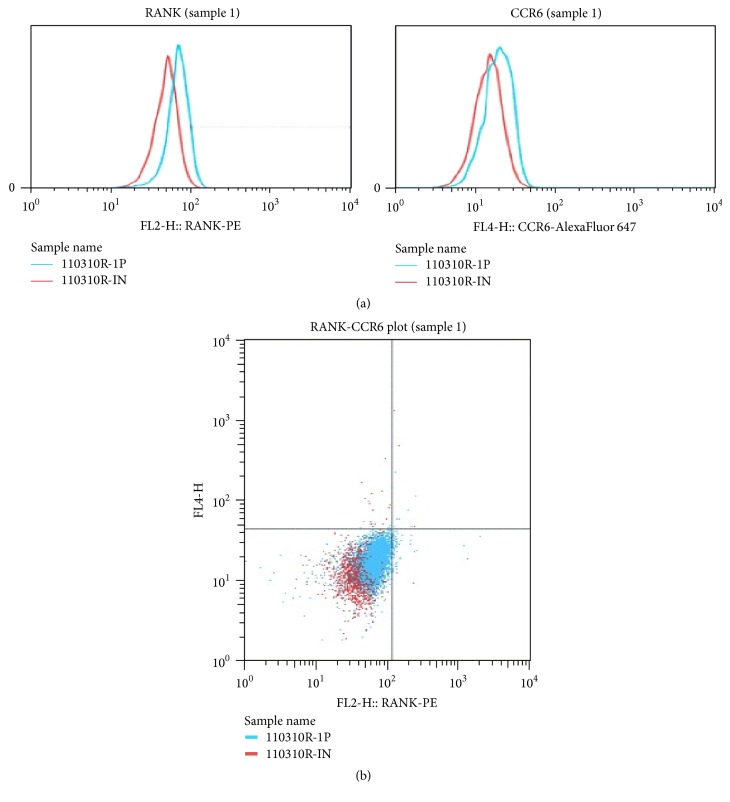
(a) Expression of RANK and CCR6 on monocytes from HV. The left figure shows RANK expression using PE. The right figure shows CCR6 expression using AlexaFluor 647. Blue: specific detection (110310R-1P) and red: negative control using nonspecific antibodies (110310R-IN). (b) Expression of RANK and CCR6 on monocytes from HV. RANK-CCR plot; *x*-axis represents RANK expression using PE. *y*-axis represents CCR expression using AlexaFluor 647. Blue: specific detection and red: negative control using nonspecific antibodies.

The high-level expression of RANK on CD14^+^ cells was correlated with that of CCR6 in HV. Figures 4(a) and 4(b) show the representative expression of RANK and CCR6 on monocytes from HV.  Table 1 presented the rates of cell subtypes in PB. As shown in Figures 4(a) and 4(b), the high-level expression of RANK on CD14^+^ cells was correlated with that of CCR6. As presented in Table 1, the rates of RANK^high^ on monocytes, CCR6^+^ on monocyte, and CCR6^+^ on RANK (–) monocytes in RA patients were significantly higher in HV, respectively.

## 4. Discussion

In this study, we demonstrated that (1) the rate of the expression of CD14^+^RANK^high^ on monocytes in untreated RA patients was elevated compared with that of those receiving treatment; (2) the rate of the expression of CD14^+^RANK^high^ on monocytes was decreased with disease improvement after treatment with DMARDs in RA patients; (3) total area of OC from PBMC of HV was correlated with MFI of RANK; (4) the high-level expression of RANK on CD14^+^ cells was correlated with that of CCR6 in HV.

From these results, osteoclastogenesis may decrease after treatment with DMARDs. Monocytes expressing both RANK and CCR6 differentiate into osteoclasts. In addition, levels of expression of RANK on monocytes were positively correlated with the level of osteoclastogenesis derived from PBMC. Thus, these monocytes play a critical role in joint destruction.

Since 2001, we have reported that the expression of RANKL on activated T cells from untreated RA patients is elevated [6]. Moreover, high-level expression of RANK on CD14^+^ cells shown in the current study may induce osteoclastogenesis via activated T cells expressing RANKL. In addition, we and other groups have demonstrated that IL-17A plays an important role inducing RANKL in osteoblasts in the pathogenesis of RA [7, 8]. Thus, RANK-RANKL system is involved in both osteoclastogenesis and the immune system [9, 10].

**Figure 5:**
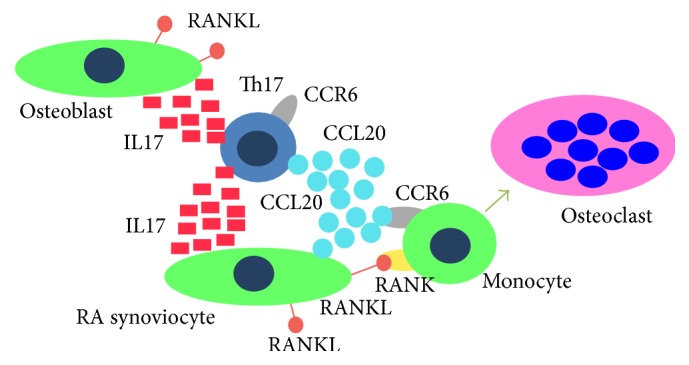
Hypothesis: CCR6^+^RANK^+^ monocytes in an RA joint.

CCR6, the gene encoding chemokine (C-C motif) receptor 6, a surface marker for Th17 cells at 6q27, is critically involved in IL-17A-driven autoimmunity in diseases and associated with RA susceptibility [3]. Th17 cells express both CCR6 and CCL20, a ligand of CCR6. In addition, CCL20 is expressed in fibroblast-like synoviocytes (FLS) from RA patients [4, 11, 12]. Thus, we hypothesized that CCR6^+^RANK^+^ monocytes migrate into the joints where CCL20 production is elevated (Figure 5). These monocytes differentiate into osteoclasts due to the presence of RANKL expressed on synoviocytes. Thus, monocytes expressing both RANK and CCR6 play a pivotal role in the joint destruction of RA. CCL20 chemokine induces osteoclast differentiation [12]. Increased levels of CCL20 are expressed in subchondral bone tissues of rheumatoid arthritis patients. CCL20 does not stimulate osteoclast formation directly.

The pathogenesis of osteoporosis remains to be elucidated. Pacifici et al. [13] reported that, in idiopathic osteoporosis, the spontaneous release of interleukin 1 from human blood monocytes reflects bone formation. Moreover, they [14] also reported that ovarian steroid treatment blocks a postmenopausal increase in blood monocyte interleukin 1 release. In these studies, monocytes* per se* may play an important role in osteoporosis. In the current study, the expression of RANK was diverse in each HV. Surprisingly, the total areas of OC from PBMC of HV were correlated with MFI of RANK. Thus, it is suggested that the high-level expression of RANK on monocytes plays a role in osteoporosis.

In summary, since the high-level expression of RANK on CD14^+^ monocytes elevated osteoclastogenesis, these cells may play a crucial role in joint destruction and osteoporosis. Monocytes expressing both RANK and CCR6 differentiate into osteoclasts. Levels of expression of RANK on monocytes were positively correlated with the level of osteoclastogenesis. Thus, it is possible that these monocytes play a crucial role in the joint destruction of RA. RANK and CCR6 expressed on monocytes may be novel targets for the regulation of bone resorption in RA patients.

## Figures and Tables

**Table 1:** Percentages of cell subtype in PB (%).

	RANK^+^ in Mo	CCR6^+^ in Mo	CCR6^+^ in RANK^−^ Mo	CCR6^+^ in RANK^+^ Mo
RA (*n* = 14)	4.6	2.8	2.3	15.0
HC (*n* = 5)	1.8	1.3	1.1	14.2
*p* value (*t*-test)	0.003	0.009	0.033	0.891

## Abbreviations

DMARDs: Disease modifying antirheumatic drugs
HV: Healthy volunteers
OA: Osteoarthritis
PBMCs: Peripheral bold mononuclear cells
RA: Rheumatoid arthritis
RANK: Receptor activator of NF-*κ*B
RANKL: TNF receptor superfamily and a receptor for the RANK ligand.
